# FXR Mediates Adenylyl Cyclase 8 Expression in Pancreatic *β*-Cells

**DOI:** 10.1155/2019/8915818

**Published:** 2019-08-14

**Authors:** Xiangchen Kong, Bingfeng Li, Yushen Deng, Xiaosong Ma

**Affiliations:** ^1^Shenzhen University Diabetes Institute, School of Medicine, Shenzhen University, Shenzhen 518060, China; ^2^College of Life Sciences and Oceanography, Shenzhen University, Shenzhen 518060, China

## Abstract

Adenylyl cyclase 8 (ADCY8) and Farnesoid X Receptor (FXR) have been identified in pancreatic *β*-cells and play important roles in insulin secretion. But the mechanisms underlying with respect to the regulation of ADCY8 expression in *β*-cells, particularly whether FXR is involved, remain unexplored. We now show that ADCY8 expression is decreased in Goto-Kakizaki (GK) rat islets compared with healthy Wistar controls. We also found that reduced ADCY8 is associated with decreased expression of FXR. Consistently, ADCY8 expression was suppressed by the knockdown of FXR in INS-1 832/13 cells, as well as the islets from FXR knockout mice. On the contrary, ADCY8 expression was increased in FXR-overexpressed INS-1 832/13 cells or in the case of FXR activation. Mechanistically, FXR directly binds to *Adcy8* promoter and recruits the histone acetyltransferase Steroid Receptor Coactivator 1 (SRC1), thereby resulting in the increased acetylation of histone H3 in *Adcy8* locus, promoting *Adcy8* gene transcription in *β*-cells. Thus, this study indicates that FXR is a critical transcription factor that mediates ADCY8 expression in pancreatic *β*-cells and has characterized the chromatin modification associated with *Adcy8* transcription.

## 1. Introduction

Adenylyl cyclase 8 (ADCY8), one member of the adenylyl cyclase family that is responsible for the generation of cAMP, has been identified in pancreatic *β*-cells [1] and implicated in the regulation of insulin secretion. It has been reported that ADCY8 mediates the effects of nutrients and insulinotropic peptide such as glucagon-like peptide 1 (GLP-1) on insulin secretion [1]. ADCY8 is activated by the increase of cytosolic calcium induced by glucose, whereas the knockdown of ADCY8 abrogates insulin secretion stimulated by glucose [2]. Moreover, the knockout of *Adcy8* impairs insulin secretion of mouse islets [2]. In addition, ADCY8 plays a central role in GLP-1 signaling pathway in rat and human pancreatic *β*-cells [3]. Notably, ADCY8 is significantly reduced in islets from diabetic Goto-Kakizaki (GK) rats [4] or in pancreatic *β*-cells at glucotoxicity [3], suggesting that the reduction of ADCY8 is associated with impaired secretory capacity of *β*-cells in diabetes.

FXR is one of the bile acid receptors and a ligand-activated transcription factor belonging to the nuclear receptor superfamily [5]. It has been found that FXR is expressed in human and rodent *β*-cells [6, 7]. The activation of FXR affects the expression of its target genes, a process via binding to FXR response elements (FXRE) and subsequent recruiting several coactivators such as SRC1 [8]. The activation of FXR by bile acids or GW4064 also enhances insulin secretion [6, 7]. However, how FXR does so, particularly the downstream target gene involved, remains largely unknown. In the present study, we demonstrated that ADCY8 is one of the target genes regulated by FXR and could be involved in FXR-enhanced insulin secretion.

## 2. Materials and Methods

### 2.1. Reagents and Antibodies

GW4064 (Cat. G5172), protease inhibitor cocktail (Cat. P8340), and *β*-actin antibody (Cat. A5441) were purchased from Sigma (St. Louis, MO, USA). Collagenase P (Cat. 11213865001) was provided by Roche Molecular Biochemicals (Indianapolis, IN, USA). Adenylyl cyclase VII antibodies (Cat. sc-32131 and ab175874) were purchased from Santa Cruz Biotechnology (CA, USA) and Abcam (Cambridge, MA, USA), respectively. Insulin (Cat. 3041) and GAPDH (Cat. 5174) antibodies were obtained from Cell Signaling (Danvers, MA, USA). FXR (Cat. 417200) and Acetyl-Histone H3 (Cat. 06-599) and Alexa-Fluor-labeled secondary antibodies (Cat. A11035 and A11029) were provided by Thermo Fisher Scientific (Waltham, MA, USA). SRC1 (Cat. ab2859) antibody was purchased from Abcam. Rat (Cat. 80-INSRTU-E10) and Mouse (Cat. 80-INSMSU-E10) Insulin Ultrasensitive ELISA Kit was purchased from ALPCO Diagnostics (Salem, NH, USA). Nuclear-cytosol extraction kit (Cat. P1200) was purchased from Applygen Technologies (Beijing, China). cAMP assay kit (Cat. ADI-900-066) was provided by Assay Designs (Ann Arbor, MI, USA).

### 2.2. Animals

Wistar and Goto-Kakizaki (GK) rats (blood glucose concentration is more than 15 mmol/l) were purchased from Shanghai Laboratory Animal Company (SLRC, Shanghai, China). Zucker Diabetic Fatty (ZDF) and control rats were obtained from Beijing Vital River Laboratory Animal Technology (China). FXR knockout mice (C57BL/6) were kindly provided by Prof. Youfei Guan from Dalian Medical University (Dalian, China). Experiments were performed with male wild-type and FXR knockout mice, aged 16-20 weeks. Rat and mouse islets were isolated and cultured as described [9]. The animal procedures were performed according to the Principles of Laboratory Animal Care and approved by the Shenzhen University Animal Care Committee.

### 2.3. Cell Lines

Rat INS-1 832/13 cells were maintained in RPMI 1640 medium as we reported previously [10]. shRNAs, designed to knockdown gene expression of FXR or SRC1, were purchased from GE Dharmacon. INS-1 832/13 cells were transduced with either scramble RNA or shRNA and were selected with puromycin. To generate a cell line with an overexpression of FXR, rat *Nr1h4* coding sequence was cloned into pMX-puro vector plasmid using primers: forward: 5′-CGGGATCCATGAATCTGATTGGGCCCTC-3′, reverse: 5′-AAGGAAAAAAGCGGCCGCTCACTGCACATCCCAGATCT-3′. Then, INS-1 832/13 cells were transduced with either pMX-puro vector or pMX-puro FXR plasmid and selected with puromycin.

### 2.4. Western Blot

Total protein was extracted from INS-1 832/13 cells and islets using RIPA lysis buffer supplemented with protease inhibitor cocktail. Cytosolic and nuclear proteins were prepared using Wistar and GK rat pancreases with a nuclear-cytosol extraction kit according to the manufacturer's protocol. In brief, the tissues were lysated in cytosol extraction buffer A and buffer B for 10 min on ice, followed by centrifuge for 5 min at 1000 × g, 4°C. The pellet contained crude nuclei. The supernatant was transferred to a new tube and further centrifuged at 12000 × g for 5 min, 4°C. The cytosol protein in the supernatant was collected for experiments. After washing twice with cytosol extraction buffer A, crude nuclei were incubated with nuclear extraction buffer for 30 min on ice, followed by centrifuge at 12000 × g for 5 min at 4°C. Then, the supernatant containing the nuclear protein was collected and used for experiments. The protein concentration of the samples was determined by BCA method. 50 *μ*g of cell lysates was loaded and separated using 10% SDS-PAGE and transferred onto PVDF membrane. The membranes were incubated with corresponding primary antibodies overnight at 4°C and then incubated with horseradish peroxidase-conjugated secondary antibody for 2 h at room temperature. The immunoreactive bands were visualized by the GE LAS4000 imaging system. The densities of the bands were determined using Gel-Pro Analyzer 4.0 software and normalized against the level of GAPDH or *β*-actin.

### 2.5. Real-Time PCR

Total RNA was extracted from INS-1 832/13 cells and islets using Trizol reagent according to the manufacturer's instructions. 1 *μ*g RNA was used to prepare cDNA. Real-time PCR was performed with SYBR Green master mix on qTOWER2.2 real-time PCR System (Germany). The following primers were used: ADCY8 forward: 5′-GGCACCAAAGTCTTTCCG-3′, ADCY8 reverse: 5′-TGAAGGAGTTGCGTAGGG-3′; GAPDH forward: 5′-CCTTCATTGACCTCAACTAC-3′, GAPDH reverse: 5′-TCGCTCCTGGAAGATGGTGAT-3′; and *β*-actin forward: 5′-GTAAAGACCTCTATGCCAACA-3′, *β*-actin reverse: 5′-GGACTCATCGTACTCCTGCT-3′. The quantity of ADCY8 mRNA was normalized to the internal control of GAPDH or *β*-actin.

### 2.6. Immunocytochemistry

These experiments were performed as reported previously [9]. The following antibodies were used: mouse anti-FXR antibody (1 : 100) and rabbit anti-insulin antibody (1 : 100). Alexa-Fluor488-labeled goat anti-mouse IgG (1 : 100) and Alexa-Fluor 546-labeled goat anti-rabbit IgG (1 : 100).

### 2.7. Chromatin Immunoprecipitation (ChIP) Assay

ChIP assays were performed using a ChIP assay kit (Millipore) according to the manufacturer's instructions. Chromatin was immunoprecipitated with antibodies (2 *μ*g) against FXR or acetylated histone 3 or SRC1, respectively. Final DNA extractions were sequenced between -1845 bp and -1722 bp in the *Adcy8* promoter with the following primers: forward 5′-GTGCCTTAAAGGACTTTCCC-3′; reverse 5′-AGCGTTCTCAGGGTTATTT-3′.

### 2.8. Construction of Luciferase Plasmid and Promoter Reporter Assay

Rat promoter fragments of *Adcy8* (−1908 bp to +55 bp) were amplified by PCR using primers: forward: 5′-CGAGCTCCTTCCTTGAGCGATCTTTG-3′, reverse: 5′-CCCAAGCTTGGTTCCTTCCTGGTTGTG-3′. The product was cloned into pGL3-basic luciferase reporter vector (Promega). For the determination of *Adcy8* promoter activity, 293T cells were transfected with pGL3-Adcy8 and Renilla luciferase plasmid, followed by treatment with 5 *μ*M GW4064 for 24 h. The promoter activity was determined using the Dual-Luciferase Reporter Assay Kit (Promega) according to the manufacturer's instructions. The plasmid expressing Renilla luciferase was used for normalization of luciferase activity.

### 2.9. Insulin Secretion

Insulin secretion of INS-1 832/13 cells was assayed as published [9]. Cells were preincubated in KRB buffer for 1 h at 37°C. Then, the cells were stimulated with 16.8 mM glucose alone or in the presence of 10 *μ*M forskolin for 30 min, as indicated, respectively. The level of insulin was determined using an Insulin Ultrasensitive ELISA Kit (ALPCO Diagnostics, Salem, NH). Insulin levels (ng/ml) were normalized against islet number or total cellular protein content in mg.

### 2.10. Determination of cAMP Levels

The scramble and shFXR INS-1 832/13 cells or islets from wild-type and FXR knockout mice were seeded in six-well plates and cultured in medium containing 5.5 mmol/l glucose medium for 24 h. The cells were then incubated for 30 min in KRB buffer containing 16.8 mmol/l glucose in the presence or absence of 10 *μ*M forskolin. Intracellular cAMP levels were determined using an ELISA kit according to the manufacturer's instructions.

### 2.11. Statistical Analyses

Data are presented as mean ± SEM for the indicated number of experiments (*n*). Statistical significance was evaluated using the independent *t*-test or one-way ANOVA with the least significant difference (LSD) post hoc test. Data were considered significant when *P* < 0.05.

## 3. Results

### 3.1. Reduced ADCY8 and FXR Expression in *β*-Cells from Diabetic GK Rats

**Figure 1:**
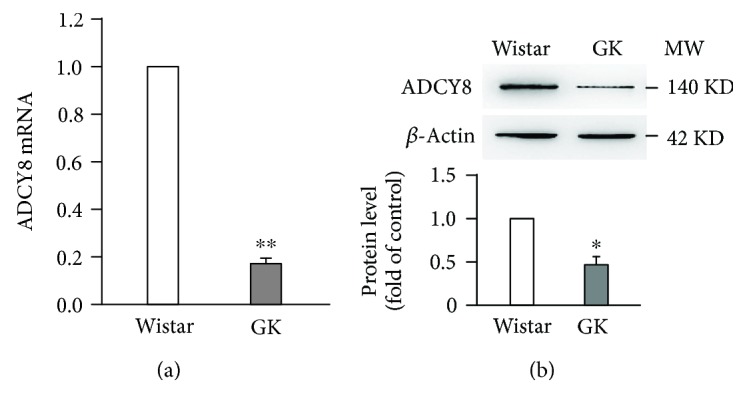
ADCY8 expression is decreased in GK rat islets. (a) mRNA level of ADCY8 in Wistar and GK islets. Values represent as means ± SEM. *n* = 5; ^∗∗^ *P* < 0.01. (b) Representative blots of ADCY8 protein expression of three independent experiments in Wistar and GK islets. Data are means ± SEM. ^∗^ *P* < 0.05.

We examined ADCY8 expression in islets from GK rats and found ADCY8 mRNA (Figure 1(a)) and protein (Figure 1(b)) reduced by ~80% (*P* < 0.01) and ~50% (*P* < 0.05), respectively, as compared with those of the Wistar controls, which are consistent with a previous study [4]. These results were also confirmed in ZDF rat islets (Supplementary Figure 1).

**Figure 2:**
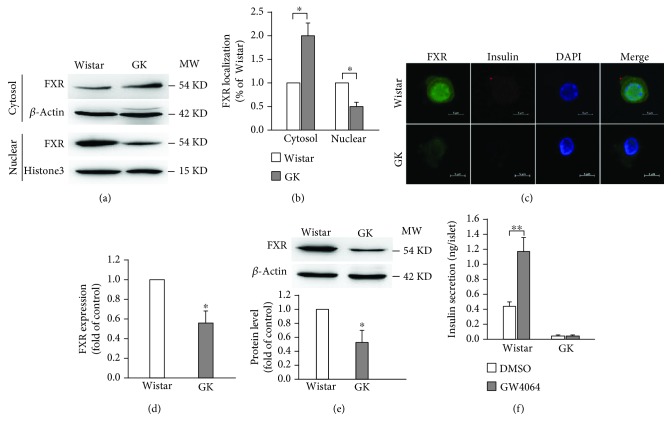
Expression of FXR in islets from Wistar and GK rats. (a, b) Cytosol and nuclear proteins extracted from Wistar and GK rat pancreases were subjected to western blot analysis. Histone3 and *β*-actin were used as nuclear and cytosol internal control, respectively. Data are means ± SEM. *n* = 3; ^∗^ *P* < 0.05. (c) Representative immunostaining images for FXR (green), insulin (red), DAPI (blue), and merge of the three in single *β*-cells isolated from Wistar and GK rats; *n* = 4. Scale bars represent 5 *μ*m. (d) Statistical analysis of FXR expression in *β*-cells isolated from Wistar and GK rats. Data are means ± SEM. *n* = 4; ^∗^ *P* < 0.05. (e) FXR protein expression in Wistar and GK rat islets. Data represent means ± SEM. *n* = 3; ^∗^ *P* < 0.05. (f) Insulin secretion in islets isolated from Wistar and GK rats. The islets were treated with 16.8 mM glucose in the presence of DMSO or 5 *μ*M GW4064 for 30 min. Data are means ± SEM. *n* = 4; ^∗^ *P* < 0.05.

To clarify whether the expression of FXR is altered in diabetes, we, firstly, investigated the intracellular localization of FXR in Wistar and GK pancreases. The results revealed that the nuclear localization of FXR was reduced in GK rat pancreases, accompanied by increased cytoplasmic distribution, as compared with that of Wistar rats (Figures 2(a) and 2(b)). In order to eliminate the interference from other cells in the pancreas, we then detected FXR expression in single *β*-cells from Wistar and GK rat islets. The data showed that FXR expression is obviously decreased in *β*-cells from GK rats, as compared with that of Wistar rats (Figures 2(c) and 2(d)). Moreover, this observation was further confirmed in islets from Wistar and GK rats by western blot (Figure 2(e)). Consistently, FXR agonist GW4064-stimulated insulin secretion was diminished in islets from GK rats, albeit GW4064 induced a ~2.6-fold increase of insulin secretion in Wistar rats (Figure 2(f)).

### 3.2. FXR Regulates ADCY8 Expression in *β*-Cells

**Figure 3:**
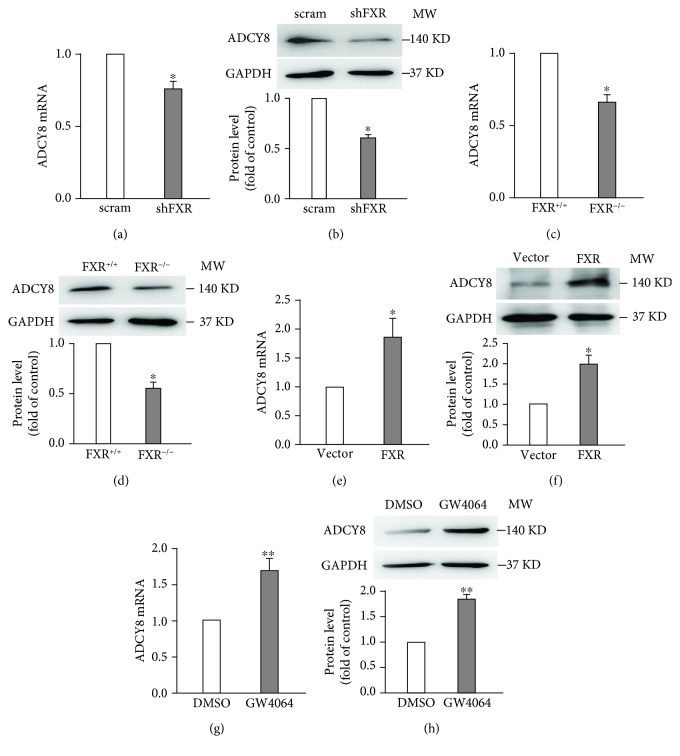
FXR regulates ADCY8 expression in pancreatic *β*-cells. mRNA (a) and protein (b) level of ADCY8 in FXR knockdown INS-1 832/13 cells. Data are means ± SEM. *n* = 4‐5; ^∗^ *P* < 0.05. mRNA (c) and protein (d) level of ADCY8 in FXR knockout mouse islets. 100-150 islets were used in each experiment. Data represent means ± SEM. *n* = 3‐5; ^∗^ *P* < 0.05. mRNA (e) and protein (f) expression of ADCY8 in FXR-overexpressed INS-1 832/13 cells. Data are means ± SEM. *n* = 4; ^∗^ *P* < 0.05. mRNA (g) and protein (h) expression of ADCY8 in INS-1 832/13 cells after 24 h treatment with 5 *μ*M GW4064. Data are means ± SEM. *n* = 4‐7; ^∗∗^ *P* < 0.01.

To explore whether FXR affects ADCY8 expression in INS-1 832/13 cells, we first transfected INS-1 832/13 cells with scramble or shFXR and analyzed the mRNA and protein levels of ADCY8 by qPCR and western blotting. As shown in Figures 3(a) and 3(b), the knockdown of FXR led to a substantial reduction of ADCY8 mRNA (Figure 3(a)) and protein (Figure 3(b)) levels. The findings in insulin-secreting cell lines are further confirmed by the observations made in primary islets from FXR^+/+^ and FXR^−/−^ mice. Thus, FXR^−/−^ islets had substantially lower ADCY8 mRNA (Figure 3(c)) and protein (Figure 3(d)) levels than FXR^+/+^ islets. To further confirm that ADCY8 is regulated by FXR, we performed qPCR and western blot in INS-1 832/13 cells transduced with either pMX-puro vector or pMX-puro FXR plasmid. The results showed that FXR-overexpressed INS-1 832/13 cells displayed significant higher mRNA (Figure 3(e)) and protein (Figure 3(f)) levels of ADCY8 than vector controls. This is consistent with the observation in INS-1 832/13 cells treated with FXR agonist GW4064 for 24 h (Figures 3(g) and 3(h)).

### 3.3. Activation of FXR Induces Direct Binding of FXR to the Promoter of ADCY8 to Promote ADCY8 Expression

**Figure 4:**
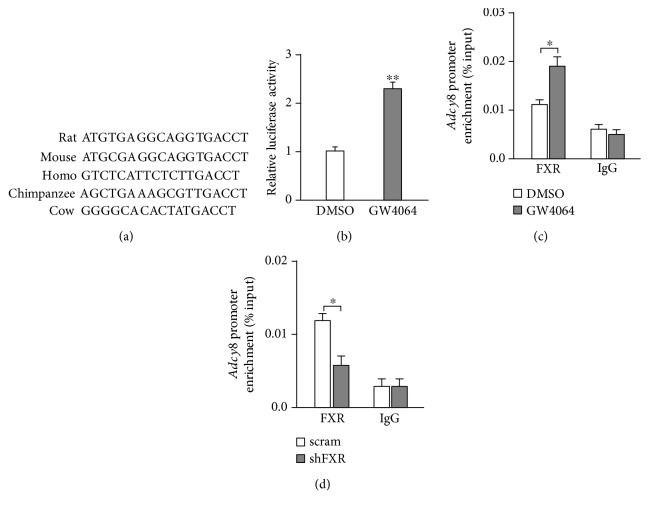
FXR directly binds to the *Adcy8* promoter. (a) A putative FXR binding site in the *Adcy8* promoter of different species. (b) 293T cells were transfected with a luciferase reporter driven by the *Adcy8* promoter, followed by treatment with 5 *μ*M GW4064 for 24 h prior to luciferase activity analysis for the reporter expression. Data are means ± SEM. *n* = 3; ^∗∗^ *P* < 0.01. ChIP analysis for FXR occupancy in the promoter of *Adcy8* in INS-1 832/13 cells treated with 5 *μ*M GW4064 for 24 h (c) or in FXR knockdown INS-1 832/13 cells (d). Data are means ± SEM of three independent experiments. ^∗^ *P* < 0.05.

**Figure 5:**
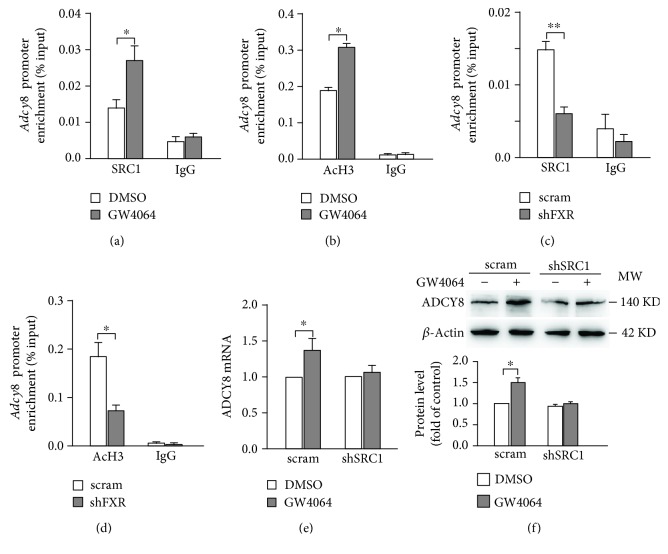
FXR mediates SRC1 binding and histone H3 acetylation at the promoter of *Adcy8*. ChIP assay was performed to detect the binding of SRC1 (a) and histone H3 acetylation (b) at the *Adcy8* promoter in INS-1 832/13 cells treated with 5 *μ*M GW4064 for 24 h. Data represent means ± SEM. *n* = 3; ^∗^ *P* < 0.05. FXR knockdown INS-1 832/13 cells were subjected ChIP assay to test SRC1 binding (c) and histone H3 acetylation (d) at the *Adcy8* promoter. Data are means ± SEM. *n* = 3; ^∗^ *P* < 0.05, ^∗∗^ *P* < 0.01. mRNA (e) and protein (f) expression of ADCY8 in scramble or SRC1 knockdown INS-1 832/13 cells treated with 5 *μ*M GW4064 for 24 h. Data are means ± SEM. *n* = 3; ^∗^ *P* < 0.05.

The data of Figures 3(a)–3(h) suggest that ADCY8 would be one of the downstream target genes regulated by FXR in *β*-cells. Analyses of DNA sequence in the *Adcy8* locus revealed a consensus “TGACCT” sequence of the FXR binding site (FXRE) in the *Adcy8* promoter that is conserved across a variety of species (Figure 4(a)), indicating that FXR is capable of binding to the promoter of *Adcy8* gene directly. To test this idea, we transfected 293T cells with ADCY8-Luc and Renilla luciferase plasmid, followed by treatment with GW4064 for 24 h. FXR activation by GW4064 significantly increased the transcription activity of the *Adcy8* gene promoter (Figure 4(b)). This is indeed consistent with the results from the ChIP assay. Thus, treatment with GW4064 increased (Figure 4(c)) whereas FXR knockdown decreased (Figure 4(d)) the binding of FXR to the *Adcy8* promoter. Our ChIP data further revealed that the treatment of INS-1 832/13 cells with GW4064 increased the recruitment of histone acetyltransferase SRC1 to the *Adcy8* promoter (Figure 5(a)), as well as the level of histone H3 acetylation (Figure 5(b)), the histone marker for active gene transcription, at the *Adcy8* locus [11]. On the contrary, FXR knockdown resulted in decreased recruitment of SRC1 (Figure 5(c)) and reduced histone H3 acetylation at the *Adcy8* locus (Figure 5(d)). Furthermore, the activation of FXR by GW4064 significantly increased ADCY8 mRNA (Figure 5(e)) and protein (Figure 5(f)) levels in scramble control cells, but not in shSRC1 INS-1 832/13 cells, demonstrating that SRC1-induced histone H3 acetylation is responsible for FXR-induced ADCY8 expression.

### 3.4. Knockdown of FXR Attenuates the Effect of Forskolin on Insulin Secretion

**Figure 6:**
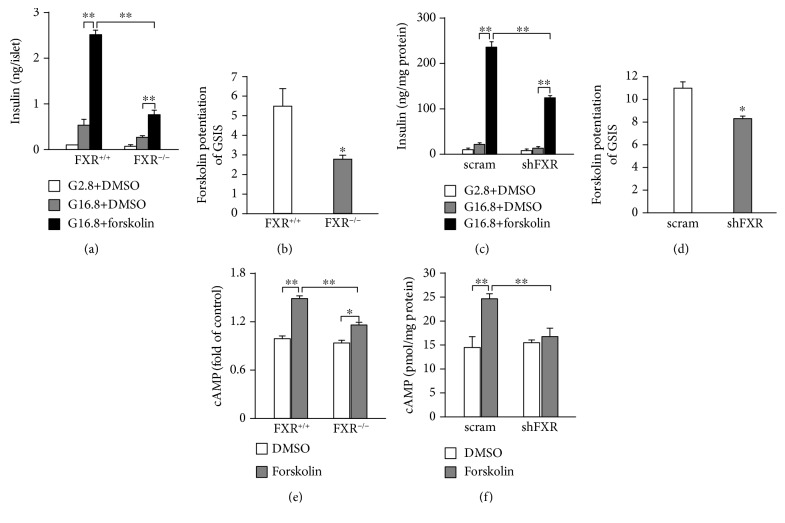
Effect of forskolin on insulin secretion and cAMP production in pancreatic *β*-cells. (a) Insulin secretion of islets isolated from FXR^+/+^ and FXR^−/−^ mice. The islets were stimulated with 2.8 mM glucose or 16.8 mM glucose in the presence or absence of 10 *μ*M forskolin for 30 min. (b) Fold increase of forskolin-potentiated insulin secretion in FXR^+/+^ and FXR^−/−^ mouse islets. Data are means ± SEM. *n* = 4; ^∗^ *P* < 0.05, ^∗∗^ *P* < 0.01. (c) Insulin secretion of scramble and shFXR INS-1 832/13 cells treated with the condition same with (a). (d) Fold increase of forskolin-potentiated insulin secretion in scramble and shFXR INS-1 832/13 cells. Data represent means ± SEM. *n* = 4‐10; ^∗^ *P* < 0.05, ^∗∗^ *P* < 0.01. cAMP production in islets from FXR^+/+^ and FXR^−/−^ mice (e) and scramble and shFXR INS-1 832/13 cells (f) incubated with 16.8 mM glucose in the presence or absence of 10 *μ*M forskolin for 30 min. Data are means ± SEM. *n* = 3 of each group. ^∗^ *P* < 0.05, ^∗∗^ *P* < 0.01.

We next explored whether FXR affects the stimulatory effects of cAMP on glucose-stimulated insulin secretion (GSIS). To this end, we treated mouse islets and INS-1 832/13 cells with forskolin (10 *μ*mol/l), a general activator of adenylyl cyclase, in KRB buffer containing 16.8 mmol/l glucose for 30 min. As shown in Figures 6(a) and 6(b), the treatment with forskolin resulted in ~5.5-fold increase of GSIS in FXR^+/+^ islets. Strikingly, however, the effect of forskolin was attenuated (*P* < 0.01) in FXR^−/−^ islets, a ~2.8-fold stimulation in this case (Figures 6(a) and 6(b)). Similar results were also obtained from scramble and shFXR INS-1 832/13 cells (Figures 6(c) and 6(d)). In addition, this phenomenon was corroborated by treatment of scramble and shFXR INS-1 832/13 cells with 10 nM GLP-1 for 30 min (Supplementary Figure 2). We reasoned that the decreased effect of forskolin would be attributed to the reduced ability of forskolin to increase intracellular cAMP levels in FXR knockout or knockdown *β*-cells. To test this idea, we determined the cytoplasmic cAMP in islets and INS-1 832/13 cells. The cells were treated with 16.8 mM glucose in the presence or absence of 10 *μ*mol/l forskolin for 30 min. As shown in Figure 6(e), forskolin induced a ~1.5-fold increase of cAMP in FXR^+/+^ islets, which is largely decreased in FXR^−/−^ islets. The similar results were also observed in scramble and shFXR INS-1 832/13 cells (Figure 6(f)).

## 4. Discussion

In the present study, we demonstrate, for the first time, that ADCY8 expression is regulated by FXR in *β*-cells. We also show that FXR-regulated transcription of *Adcy8* involves recruiting SRC1 and thus enhances histone H3 acetylation at the promoter of *Adcy8*. Furthermore, our data reveal that ADCY8 expression is depressed in islets of diabetic rats. This may due to the decreased expression of FXR in diabetic *β*-cells.

It is established that ADCY8 is important for the generation of cAMP and plays a critical role in GSIS [3, 12]. However, how ADCY8 expression is regulated is less clear. We demonstrate that *Adcy8* is one of the downstream target genes of FXR in *β*-cells. This notion is supported by five pieces of evidence. First, there is the FXR binding site (FXRE) at the *Adcy8* promoter (Figure 4(a)). Stimulation with FXR agonist GW4064 promoted whereas knockdown of FXR curtailed FXR binding to the *Adcy8* promoter (Figures 4(c) and 4(d)). Second, the activation of FXR by GW4064 increased ADCY8 expression at mRNA (Figure 3(g)) and protein (Figure 3(h)) levels. Third, the knockdown of FXR by shRNA resulted in reduced ADCY8 expression (Figures 3(a) and 3(b)). Fourth, ADCY8 expression was substantially decreased in FXR^−/−^ islets (Figures 3(c) and 3(d)). Fifth, the knockout or knockdown of FXR reduced forskolin-induced production of cAMP (Figures 6(e) and 6(f)) and forskolin-potentiated GSIS (Figures 6(a)–6(d)). The activation of FXR increases the recruitment of the epigenetic regulator histone acetyltransferase SRC1 (Figure 5(a)) and consequently enhances histone H3 acetylation at the *Adcy8* locus (Figure 5(b)). This would lead to increased expression of *Adcy8* gene, given that the acetylation of nucleosomal histones increases the accessibility of DNA to transcription factors and leads to increased transcription at the target DNA locus [13]. Consistently, the lack of effect of GW4064 on ADCY8 expression (Figures 5(e) and 5(f)) in shSRC1 cells again indicates that SRC1 is responsible for these effects of FXR. Thus, our results unravel a novel molecular mechanism of regulation of ADCY8 expression in pancreatic *β*-cells: FXR-mediated increase of ADCY8 expression via SRC1 and histone H3 acetylation. Further studies are required for the elucidation of the crosstalk of FXR-mediated cascade and GLP-1 signaling in *β*-cells.

FXR is the bile acid nuclear receptor and expressed in *β*-cells [6, 7]. Previous studies have demonstrated that FXR plays an important role in the regulation of glucose and lipid metabolism in rodent models [14, 15]. But the expression of FXR in diabetic pancreatic *β*-cells remains largely unknown. Our data suggest that FXR expression is suppressed in *β*-cells of GK rats (Figures 2(c)–2(e)), which is similar to a previous study that FXR expression is decreased in the livers of diabetic rats [16]. This appearance is supported by the observation that the stimulation of GW4064 on insulin secretion is almost disappeared in GK rat islets (Figure 2(f)). The reduced FXR protein level in the nucleus of GK rat pancreases (Figures 2(a) and 2(b)) could be attributed to the decreased FXR expression in *β*-cells of GK rats, whereas increased FXR protein level in the cytoplasm in the pancreases of GK rats (Figures 2(a) and 2(b)) may be caused by aberrant expression or distribution of FXR in other cells of the pancreas, since FXR is also expressed in exocrine cells of the pancreas [17]. Apparently, our results seem to contrast with a previous study that FXR is predominantly localized in the nucleus in obese mice [18]. It is noteworthy that GK rats used in our experiments are nonobese diabetic model. Therefore, this discrepancy may be resulted from different experimental models. The reduced FXR expression in *β*-cells would result in decreased transcriptional activity of target genes, such as *Adcy8*, regulated by FXR.

In summary, the results of this study provide new insight into the molecular mechanisms underlying *Adcy8* expression, thus FXR-SRC1-mediated transcription of *Adcy8* in *β*-cells.

## Data Availability

The data used to support the findings of this study are available from the corresponding author upon request.

## Supplementary Materials

Supplementary Materials Supplementary Figure 1: ADCY8 expression in islets of ZDF rats. Supplementary Figure 2: GLP-1-potentiated insulin secretion in FXR knockdown INS-1 832/13 cells.
