# Supplementary material for: FXR Mediates Adenylyl Cyclase 8 Expression in Pancreatic β-Cells
Source: J Diabetes Res. 2019 Aug 14;2019:8915818. doi: 10.1155/2019/8915818 (PMC6710725; doi:10.1155/2019/8915818)
Supplement: Supplementary Materials — Supplementary Figure 1: ADCY8 expression in islets of ZDF rats. Supplementary Figure 2: GLP-1-potentiated insulin secretion in FXR knockdown INS-1 832/13 cells. [file 8915818.f1.pdf]

Supplementary materials

Supplementary Figure Legends

Supplementary Figure 1. ADCY8 expression in islets of ZDF rats.

Representative blots of ADCY8 protein expression of three independent experiments in control and ZDF rat islets. Data are means ± SEM. *p<0.05.

Supplementary Figure 2. GLP-1 potentiated insulin secretion in FXR knockdown INS-1 832/13 cells.

Scramble and shFXR INS-1 832/13 cells were stimulated with 2.8 mM glucose or 16.8 mM glucose in the presence or absence of 10 nM GLP-1 for 30 min. Data are means ± SEM. n=4. **p<0.01.

Suppl.Fig.1

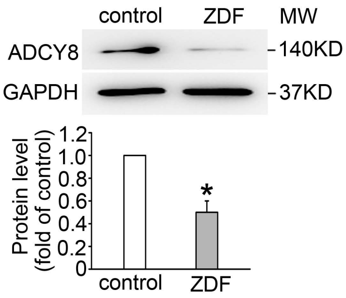

control
ZDF
MW
ADCY8
140KD
GAPDH
37KD
Protein level (fold of control)
1.2
1.0
0.8
0.6
0.4
0.2
0
*
control
ZDF

Suppl.Fig.2

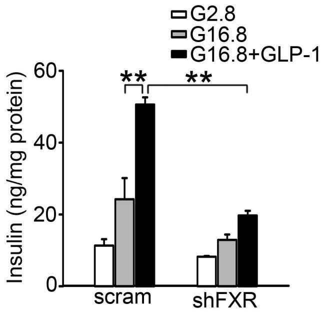
G2.8
G16.8
G16.8+GLP-1
Insulin (ng/mg protein)
60
40
20
0
**
**
scram
shFXR
